# Mice Carrying a Hypomorphic Evi1 Allele Are Embryonic Viable but Exhibit Severe Congenital Heart Defects

**DOI:** 10.1371/journal.pone.0089397

**Published:** 2014-02-27

**Authors:** Emilie A. Bard-Chapeau, Dorota Szumska, Bindya Jacob, Belinda Q. L. Chua, Gouri C. Chatterjee, Yi Zhang, Jerrold M. Ward, Fatma Urun, Emi Kinameri, Stéphane D. Vincent, Sayadi Ahmed, Shoumo Bhattacharya, Motomi Osato, Archibald S. Perkins, Adrian W. Moore, Nancy A. Jenkins, Neal G. Copeland

**Affiliations:** 1 Institute of Molecular and Cell Biology, Singapore, Singapore; 2 Welcome Trust Centre for Human Genetics, Oxford, United Kingdom; 3 Cancer Science Institute, Singapore, Singapore; 4 MYSM School of Medicine, Yale University School of Medicine, New Haven, Connecticut, United States of America; 5 Department of Pathology and Laboratory Medicine, University of Rochester Medical Center, Rochester, New York, United States of America; 6 RIKEN Brain Science Institute, 2-1 Hirosawa, Wako-shi, Saitama, Japan; 7 Department of Development and Stem Cells, Institut de Génétique et de Biologie Moléculaire et Cellulaire, CNRS UMR 7104, Inserm U964, Université de Strasbourg, Illkirch, France; UT-Southwestern Med Ctr, United States of America

## Abstract

The ecotropic viral integration site 1 (Evi1) oncogenic transcription factor is one of a number of alternative transcripts encoded by the Mds1 and Evi1 complex locus (Mecom). Overexpression of Evi1 has been observed in a number of myeloid disorders and is associated with poor patient survival. It is also amplified and/or overexpressed in many epithelial cancers including nasopharyngeal carcinoma, ovarian carcinoma, ependymomas, and lung and colorectal cancers. Two murine knockout models have also demonstrated Evi1's critical role in the maintenance of hematopoietic stem cell renewal with its absence resulting in the death of mutant embryos due to hematopoietic failure. Here we characterize a novel mouse model (designated Evi1^fl3^) in which Evi1 exon 3, which carries the ATG start, is flanked by loxP sites. Unexpectedly, we found that germline deletion of exon3 produces a hypomorphic allele due to the use of an alternative ATG start site located in exon 4, resulting in a minor Evi1 N-terminal truncation and a block in expression of the Mds1-Evi1 fusion transcript. Evi1^δex3/δex3^ mutant embryos showed only a mild non-lethal hematopoietic phenotype and bone marrow failure was only observed in adult Vav-iCre/+, Evi1^fl3/fl3^ mice in which exon 3 was specifically deleted in the hematopoietic system. Evi1^δex3/δex3^ knockout pups are born in normal numbers but die during the perinatal period from congenital heart defects. Database searches identified 143 genes with similar mutant heart phenotypes as those observed in Evi1^δex3/δex3^ mutant pups. Interestingly, 42 of these congenital heart defect genes contain known Evi1-binding sites, and expression of 18 of these genes are also effected by Evi1 siRNA knockdown. These results show a potential functional involvement of Evi1 target genes in heart development and indicate that Evi1 is part of a transcriptional program that regulates cardiac development in addition to the development of blood.

## Introduction

The complexity of an organism is defined not only by the number of its genes, but also how expression of these genes is controlled. This also includes several post-transcriptional events that control protein production, including alternative splicing, translational repression, microRNA-induced mRNA degradation, and the regulated generation of distinct gene products through the alternative use of translational initiation sites. These various mechanisms provide a tremendous diversity of protein sequence, structure and function [1], [2]. Much improvement has been made in defining the molecular basis of these regulations. However, it remains a major challenge to integrate this knowledge into a complete understanding of the resulting physiological functions, in normal and pathological conditions.

The MDS1 and EVI1 complex locus (MECOM) contains several transcription start sites and alternative splice options. It produces multiple transcripts coding for nuclear transcription factors. One of its major gene products is ecotropic viral integration site 1 (EVI1), an oncogenic zinc finger transcription factor (TF) whose overexpression in myeloid disorders such as acute and chronic myeloid leukemia (AML and CML), and myelodysplastic syndrome (MDS) has been extensively studied and correlated with poor patient survival [3]–[5]. Amplification and/or overexpression of EVI1 have also been observed in multiple epithelial cancers, including nasopharyngeal carcinoma, ovarian carcinoma, ependymomas, and lung and colorectal cancers [6]–[11]. In addition, EVI1 controls several aspects of embryonic development including hematopoiesis where it has been shown to be important for hematopoietic stem cell (HSC) renewal [12] and angiogenesis [13]. The most oncogenic human MECOM isoform, EVI1, encodes a 1051 amino acid protein containing two zinc finger domains, a central transcriptional repression domain and an acidic C-terminal region [5], [14], [15]. The seven zinc finger domains located in the N-terminus are known to bind to a GATA-like consensus motif [13], [16]–[19], while the three zinc finger domains in the C-terminus bind to an ETS-like motif [16], [20]. Additional alternative splicing of MECOM in human and mouse produces, amongst others, two major isoforms, EVI1δ324 and MDS1-EVI1 [5], [14], [15], [21]. MDS1-EVI1 is a larger *MECOM* variant. Although *MDS1* was originally described as a distinct gene, it is now recognized to be an alternative transcription start site and part of the *MECOM* locus. MDS1-EVI1 contains a 188 amino acid extension at its N-terminus, adding the so-called PR domain, which is a derivative of the SET domain [5], [14], [15], [22]. Several lines of evidence suggest that the form of EVI1 lacking the PR domain and MDS1-EVI1 display opposite functions. The shorter isoform (EVI1) acts as an aggressive oncogene while expression of the longer isoform (MDS1-EVI1) is linked to good prognosis in cancer [23]–[25]. MDS1-EVI1 was also recently described as a regulator of long term HSC repopulating activity [21]. Another important MECOM isoform, called EVI1δ324, resembles EVI1 but lacks zinc fingers motifs 6 and 7, which prevents its binding to GATA-like sites. Additional alternative splicing lead to the deletion of 9aa in the repressor domain of EVI1, MDS1-EVI1, or EVI1δ324 [14], [26]–[28], thus producing additional isoforms.

The exact physiological roles of these various *MECOM* products remain to be characterized. Two mouse knockout models have been previously reported that target *MECOM*. The first one was produced by deletion of Evi1 exon 7 [13], [29] while the second represents a conditional deletion of exon 4 [12]. For both alleles, homozygous Evi1^-/-^ mice resulted in the deletion of both Evi1 and Mds1-Evi1 transcripts. Both phenotypes showed embryonic lethality and impairment of hematopoiesis due to the loss of HSC renewal ability.

In this study, we analyzed a new conditional mutant allele of Mecom that was produced by flanking Evi1 exon 3, also Mds1-Evi1 exon 4, with loxP sites. The removal of Evi1 exon 3 is predicted to generate a frame shift mutation that would block the translation of Mds1-Evi1 protein. As Evi1 and Evi1δ324 both have translational initiation site located in exon3, it was also predicted that their protein expression would be blocked. However, Evi1 and Evi1δ324 proteins are produced in Evi1^δex3/δex3^ tissues, likely due to an alternative translation start site located in exon 4. Thus, only the Mds1-Evi1 isoform is fully disrupted in Evi1^δex3/δex3^ mice. Evi1^δex3/δex3^ animals do not die in utero and display a different phenotype compared to exon 4 and 7 knockout mice. The analysis of this new hypomorphic exon 3 Evi1 allele has uncovered novel physiological functions for MECOM in the formation of the circulatory system and provided a better understanding of the function of the various MECOM transcripts.

## Experimental Procedures

### Animals

The Institute of Molecular and Cell Biology Animal Care and Use Committee approved all animal protocols used in this study. The Evi1 exon 3 floxed allele, *Evi1^fl3^* [21], was maintained in a pure C57BL/6 background. After crossing to a β-actin-Cre deleter strain to generate the *Evi1^δex3^* null allele, *Evi1^δex3^* bearing mice were a mixture of strains 129/Sv and C57BL/6. They were made congenic on a C57BL/6 background over the course of the study, with no observed change in the experimental results. Mice were genotyped by PCR using primers F1 (5′- GGAGTGTTAAGCTTGAATTCC-3′), F2 (5′-GAAGAGCTCTTGCTGTTCATG-3′), and R7 (5′- CAGCTTAGACCTCAGCTAAC-3′). F2 and R7 were used to discriminate between the *Evi1^fl3^* (375 bp) and wild type (269 bp) alleles. F1 and R7 were used to detect the *Evi1^δex3^* allele (125 bp) (Fig. S1A,B in File S1). Vav-iCre was genotyped using Cre-F (5′-GCCTGCATTACCGGTCGATGCAACGA-3′) and Cre-R (5′-GTGGCAGATGGCGCGGCAACACCATT-3′) primers (700 bp amplicon). Blood was obtained by retro-orbital bleeding for adult mice, and by decapitation for embryos. Blood counts were performed with a Hemavet 950 device.

### Quantitative real time RT-PCR (qRT-PCR)

RNA was isolated from mouse tissues using Trizol and an RNeasy Mini Kit (Qiagen), and 0.5–2 µg were used for cDNA synthesis (*SuperScript III* First-Strand Synthesis; Invitrogen) with oligodT. qPCR was performed with the ABI-Prism 7500 (Applied Biosystems), SYBR green Master Mix, and primers designed with Primer Express Software v2.0 (Applied Biosystems). A primer list is provided in File S1. We used the 2^−δδCt^ method [30] to calculate the fold change of expression. Relative expression was normalized to *Tubg1* mRNA levels.

### Protein extraction and immunoblotting

Snap frozen tissues were processed for protein extraction as previously described [31]. Immunoblotting was performed using a protocol previously described [16]. Evi1 antibody was produced in rabbits [19] and γ-tubulin antibody was from Sigma.

### HSC characterization

Hematopoietic cells were extracted from the fetal liver or bone marrow. Flow cytometric analyses and cell sorting were performed using a LSR II, a fluorescence-activated cell sorter (FACS) Vantage, or a FACSAria as previously described [32]. Antibodies were purchased from BD Biosciences: PE-conjugated anti-Gr1 (RB6-8C5), Mac-1 (M1/70), Ter119 (TER-119), CD4 (RM4-5), CD3 (145-2C11), CD8 (53–6.7), B220 (RA3-6B2), IL7Ra (SB/199), PE-Cy7-conjugated anti-c-Kit (2B8), APC-conjugated anti-Sca-1 (E13-161.7) and FITC-conjugated CD34 (RAM34). Colony forming unit-culture (CFU-C) assays, using fetal liver cells or bone marrow cells, were performed as previously described [32]. Briefly, fetal liver or bone marrow cells were cultured in 35-mm dishes in triplicate in Methocult M3231 methylcellulose medium (StemCell Tec., Vancouver, BC, Canada) supplemented with 20 ng/mL recombinant mouse IL-3, 100 ng/mL mouse SCF, 200 ng/mL mouse G-CSF and 10 ng/mL mouse EPO. Colonies were counted on day 10.

### Histology

Mice received a complete necropsy after which their tissues were fixed in 10% neutral buffered formalin overnight and embedded in paraffin. Embryos were fixed and embedded whole before sectioning. Sections of 5 µm were stained with Hematoxylin and Eosin or Giemsa.

### Magnetic Resonance Imaging and 3D reconstruction

Embryos were harvested at E15.5, euthanized and fixed in 4% paraformaldehyde (PFA) with 2 mM Gd-DTPA (gadolinium- diethylenetriaminepentacetate) as a contrast agent. Multi-embryo imaging was conducted as previously described [33]. The raw MR data were reconstructed as described previously [34]. The files were analyzed using Amira 5.3.3 software.

### In situ hybridization in embryos

Evi1 mRNA in situ hybridization was carried out using a full length Evi1 cDNA probe [35] using standard protocols. Probes were labeled using a DIG RNA Labeling Kit (Roche Applied Science, Tokyo, Japan). Detection was via an anti-DIG antibody coupled to alkaline phosphatase (Roche, Tokyo, Japan) followed by staining with BCIP-NBT (Bromo-4-chloro-3-indolyl Phosphate/Nitro Blue Tetrazolium) (Nacalai, Tokyo, Japan) as previously described [36].

## Results

### Deletion of Evi1 exon 3 results in postnatal lethality

**Figure 1:**
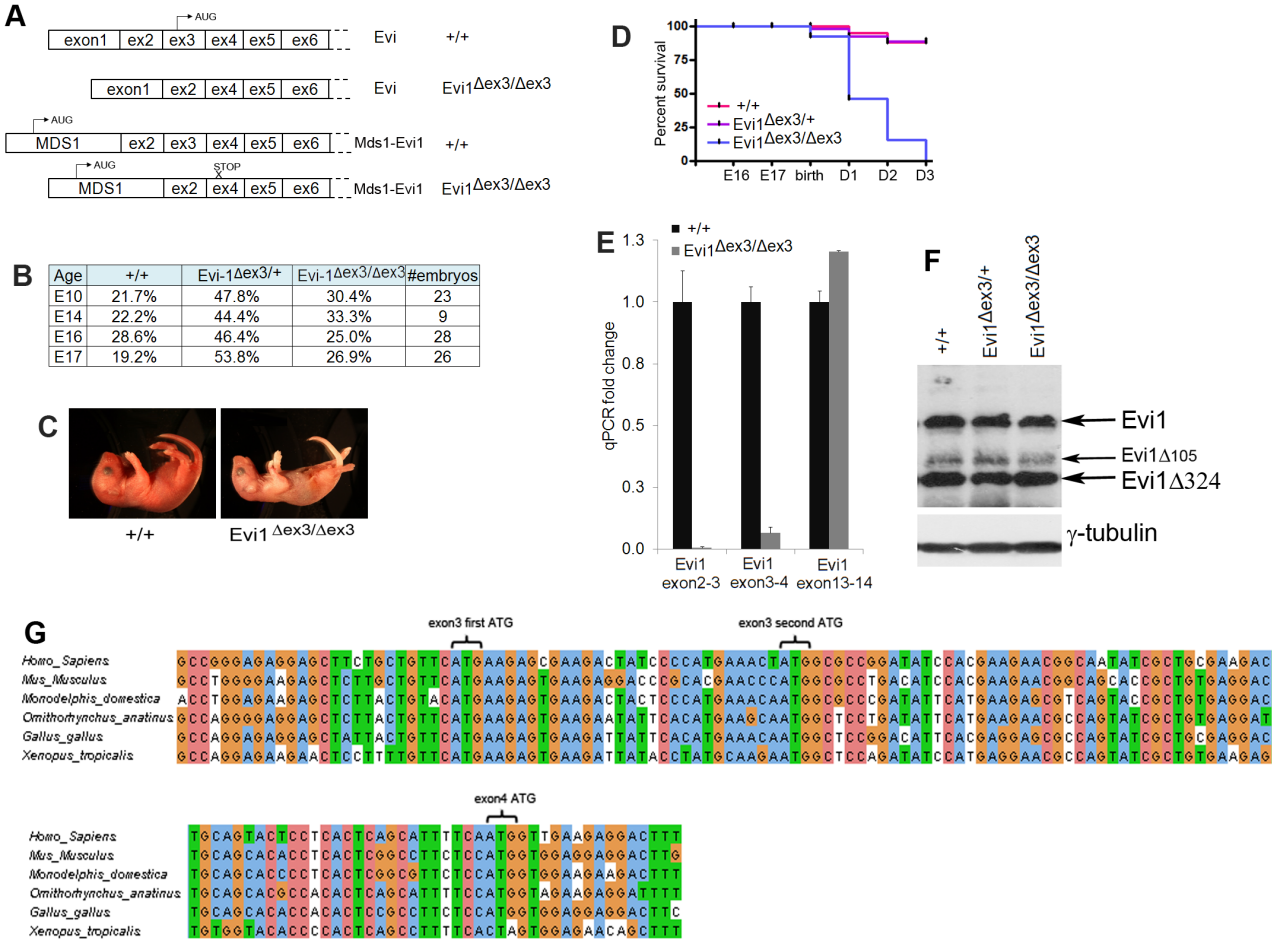
Deletion of Evi1 exon3 generates a hypomorphic allele. (A) Sequenced products obtained after 5′RACE from wild type or Evi1^δex3/δex3^ mutant embryos. (B) Table showing the fraction of embryos of each genotype detected at different stages of embryonic development. The Mendelian ratios were not affected by the Evi1 exon3 deletion. (C) Pictures of 28 hr-old littermates highlight the poor health of dying Evi1^δex3/δex3^ pups. (D) Kaplan-Meyer curves for wild type, Evi1^δex3/+^ and Evi1^δex3/δex3^ progeny indicate lethality of all Evi1^δex3/δex3^ pups by three days after birth (n = 5 to 16 per genotype). Log rank test, Chi square p value <0.0001. (E) RT-qPCR from cDNA of E14.5 embryos. The primers used amplified the regions between Evi1 exons 2 and 3, 3 and 4 or 13 and 14. Mean of three different samples per condition. The standard deviation is shown. (F) Expression of Evi1 and γ-tubulin protein products in E14.5 wild type or E17.5 Evi1^δex3/+^ and Evi1^δex3/δex3^ mutant embryos (100 µg protein/lane). (G) Nucleotide sequence of Evi1 cDNA in the exon 3 and 4 genomic region. Two ATG sites are present in exon 3 and one in exon 4. All ATGs are conserved in higher vertebrates.

Mice homozygous for an Evi1 exon 3 deletion (designed Evi1^δex3/δex3^) have recently been generated and used to access the function of Mecom in hematopoiesis ex vivo [18]. Deletion of exon3 is predicted to prematurely abrogate the expression of Mds1-Evi1 due to the presence of an out-of-frame stop codon in exon 4 (Fig. 1A). Exon 3 also encodes the ATG translation start site for Evi1 and Evi1δ324 (Fig. 1A). *Evi1^δex3^* is thus predicted to be a Mecom null allele (Fig. S1A in File S1). We therefore expected that similar to other Evi1 knockout mice [12], [13], [29], deletion of exon 3 would lead to embryonic lethality between E10.5 and E16 due to defects in HSC self-renewal and subsequent hematopoietic failure. Surprisingly, this was not the case. Homozygous Evi1^δex3/δex3^ knockout mice (Fig. S1B,C in File S1) were born with a normal Mendelian ratio (Fig. 1B). They were indistinguishable from their control littermates, there were no gross morphological defects and they were normal in size (Fig. S1D in File S1). The presence of grossly visible milk-filled stomachs a few hours after birth also attested to their ability to feed, which was confirmed by histology (Fig. S1E in File S1). However, several hours to a few days after birth, Evi1^δex3/δex3^ mice became weak, lost weight and eventually died, with no Evi1δ^ex3/δex3^ animals surviving longer than three days (Fig. 1C,D). These results suggest that Evi1^fl3^ might encode a hypomorphic allele rather than a null allele.

### Evi1^fl3^ encodes a hypomorphic allele

To determine whether Evi1^fl3^ encodes a hypomorphic allele we used 5′ RACE to confirm that exon3 was deleted from all Mecom transcripts expressed in Evi1^δex3/δex3^ embryos. We also performed RT-qPCR to quantify the level of the Mecom transcripts expressed in Evi1^δex3/δex3^ embryos using primers located in exons 2 and 3, 3 and 4 or 13 and 14. No significant amplification was detected in Evi1^δex3/δex3^ embryos using the two first sets of primers (Fig. 1E), confirming that exon3 was deleted from all Mecom transcripts in Evi1δ^ex3/δex3^ animals. Transcripts encoding Evi1 exons 13 and 14 were, however, produced at normal levels, confirming that stable Evi1 transcripts are expressed in Evi1^δex3/δex3^ embryos. Western blot analyses showed that proteins with a similar size to Evi1, Evi1δ105, and Evi1δ324 were also expressed in Evi1^δex3/δex3^ embryos (Fig. 1F). Evi1δ105 is a splice variant present in mouse but not in human tissues [37]. Deletion of exon3 thus did not appear to affect Evi1 protein translation as would have been expected by removal of exon 3. We therefore decided to look for alternative ATG translation start sites that might be located downstream of exon 3. We found a potential ATG start site in exon 4, which contains a Kozak sequence [38] and is in frame with the rest of the protein. This start site is well conserved in higher vertebrates and provides a better Kozak sequence than the start site in exon 3 (Fig. 1G, S2). The use of this alternative start site would remove 42 amino acids from the N-terminus of Evi1 including the first zinc finger motif of the proximal Evi1 zinc finger domain (Fig. S2 in File S1). Evi1δ105, an isoform specifically present in mice [37] and Evi1δ324 would be similarly affected since they share the same transcription start site as Evi1. These results support the notion that Evi1^fl3^ encodes a hypomorphic allele that results from the expression of an N-terminally truncated Evi1 protein initiated in exon 4.

### Evi1^δex3/δex3^ newborn pups have a milder hematopoietic phenotype than that observed in Evi1^δex4/δex4^ embryos

**Figure 2:**
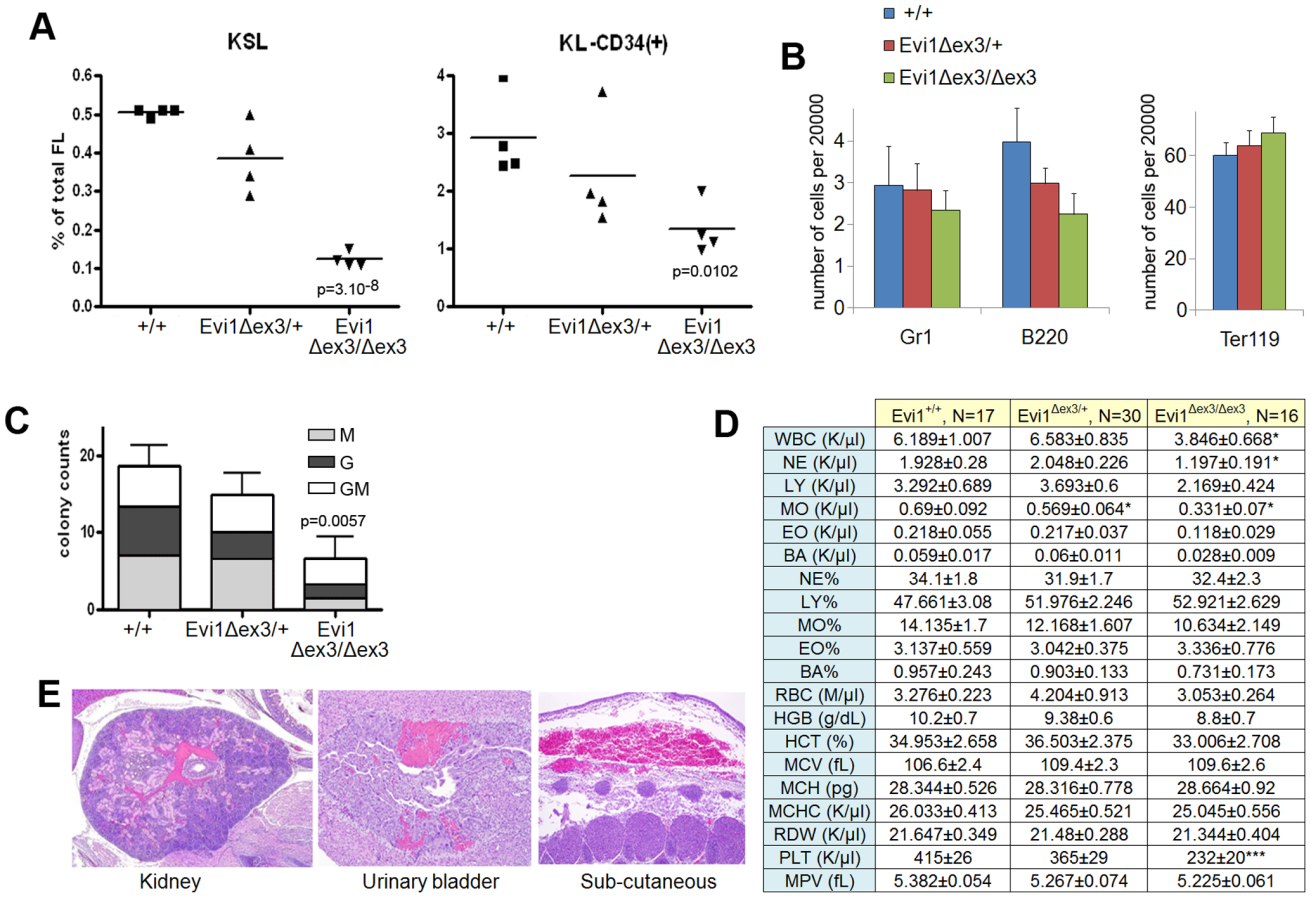
Disruption of hematopoiesis in Evi1^δex3/δex3^ newborn mice. (A,B) Flow cytometric profiles of wild type, Evi1^δex3/+^ and Evi1^δex3/δex3^ littermate fetal livers at E14.5. (A) HSC and progenitor cell subpopulations were detected by a combination of markers (KSL: c-Kit^+^, S: Sca-1^+^, L: lineage^−^, or KL-CD34^+^). We found a significant reduction of cells in the Evi1-deleted samples; p values are from an unpaired t-test between +/+ and Evi1^δex3/δex3^ fetal livers. (B) Bar graph shows the number of granulocytes (Gr1), B-lymphocytes (B220) and erythroid cells (Tert119) in fetal livers of various different genotypes. (C) Colony forming counts from cells of 3 fetal livers of each genotype at E14.5 We observed a significant reduction in colony formation between +/+ and Evi1^δex3/δex3^ fetal livers, p = 0.0057 (unpaired t-test). No BFU-E or CFU-Mix colonies were identified. (D) Hemogram results for 4 hr- to 24 hr-old wild type (N = 17), Evi1^δex3/+^, (N = 30) and Evi1^δex3/δex3^ (N = 16) littermate pups. Mean ± SEM is indicated. *p<0.05, **p<0.01, ***p<0.001, unpaired t-test. Leukocyte counts in peripheral blood and white blood cell differentials reveal a mild leucopenia in Evi1^δex3/δex3^ newborn mice. Platelet (PLT) counts and mean platelet volume (MPV) results show a mild hypoproliferative thrombocytopenia in Evi1^δex3/δex3^ pups. Normal erythrocyte counts, hemoglobin quantification and hematocrit assessment in the peripheral blood of Evi1^δex3/δex3^ animals. Mean corpuscular volume (MCV), mean corpuscular hemoglobin (MCH), mean corpuscular hemoglobin concentration (MCHC) and red cell distribution widths (RDW) are shown. (E) Hematoxylin and eosin staining of 5 µm sections of 24 hr- to 48 hr-old Evi1^δex3/δex3^ pups. Mild hemorrhages were seen in 31% of the mice (4 out of 13 pups).

The embryonic lethality in Evi1 exon 4 knockout mice has been ascribed to defective HSC self-renewal and subsequent hematopoietic failure. [12]. To determine whether *Evi1^δex3/δex3^* embryos have similar defects, we counted the number of two immunophenotypically defined HSC populations, c-Kit+, Sca-1+, lineage- (KSL) and c-Kit+, lineage-, CD34+ (KL-CD34+) cells from E14.5 wild-type, Evi1^δex3/+^ and Evi1^δex3/δex3^ fetal livers (Fig. 2A). The number of KSL HSCs and KL-CD34+ progenitor cells was significantly reduced in Evi1^δex3/δex3^ fetal livers as compared to wild type livers, while Evi1^δex3/+^ fetal livers presented an intermediate phenotype (Fig. 2A). In addition, there was a slight reduction in the number of B220+ B-lymphocytes (Fig. 2B) and colony-forming cells (Fig. 2C) in E14.4 Evi1^δex3/+^ and Evi1^δex3/δex3^ fetal livers. These results show that deletion of Evi1 exon 3 leads to a reduction in the number of HSC and progenitor cells, but this deletion does not affect the differentiation of progenitors once they are formed. This hematopoietic phenotype is milder than that described for *Evi1^δex4/δex4^* mice [12] as the HSC counts were reduced by only 76% versus 93% for *Evi1^δex4/δex4^* mice. Blood counts from Evi1^+/+^, Evi1^δex3/+^ and Evi1^δex3/δex3^ newborn animals (Fig. 2D) also showed that erythropoiesis was normal in *Evi1^δex3/δex3^* newborn animals. Mild leucopenia was however detected, which equally affected all hematopoietic compartments. Hypoproliferative thrombocytopenia was the most prominent phenotype linked to the Evi1 exon 3 deletion. Histological analyses showed that 31% of the Evi1^δex3/δex3^ pups had grossly visible focal hemorrhages in various tissues at birth (4 out of 13 pups) (Fig. 2E), while no control animals were seen with hemorrhagic lesions (0 out of 8 controls). These hemorrhages were unlikely to be the cause of embryonic lethality, however, because other genetically engineered mouse models with much lower platelet counts have been shown to survive to adulthood [39].

### Spontaneous lethal bone marrow failure in the hematopoietic compartment of Evi1^δex3/δex3^ animals

**Figure 3:**
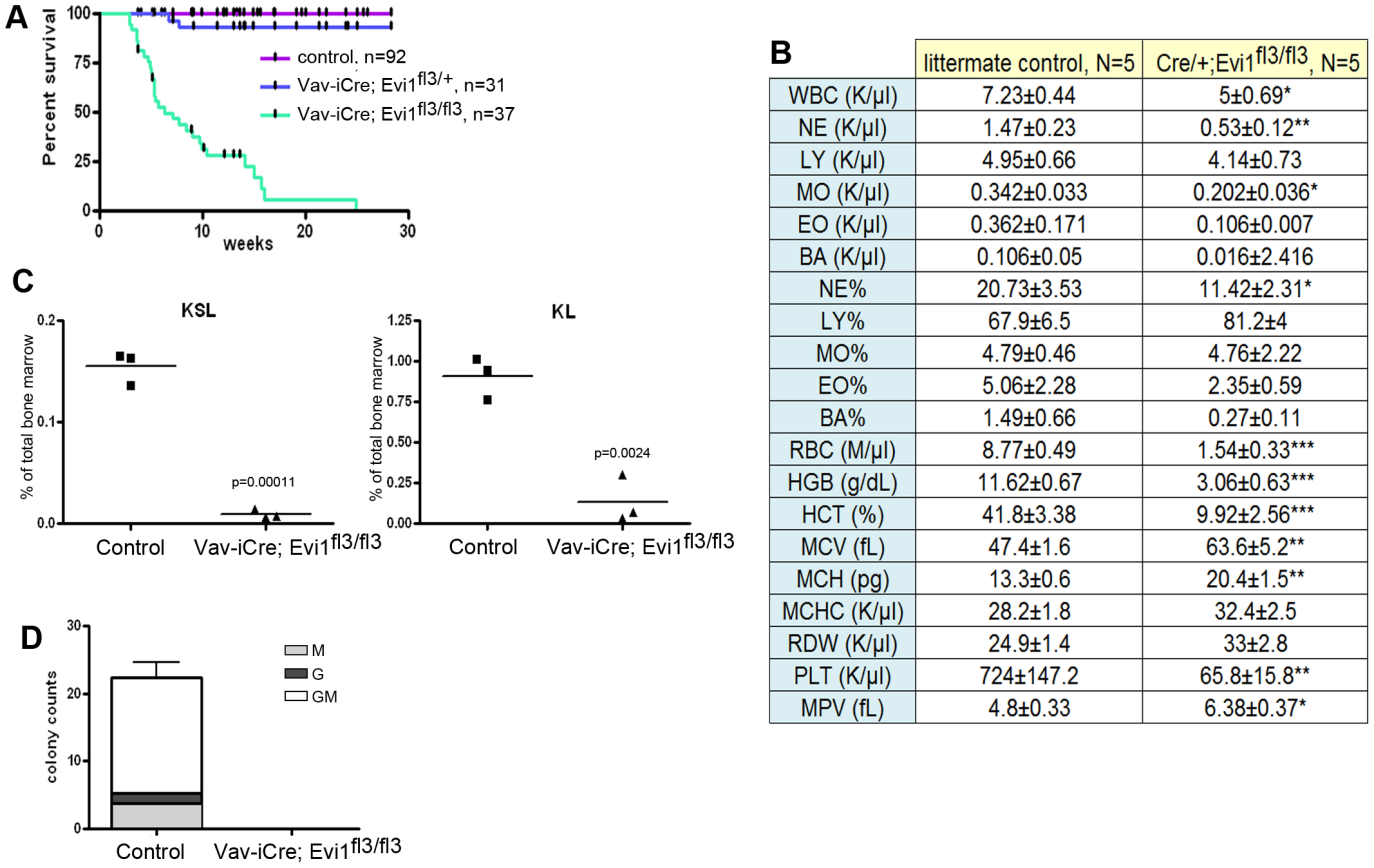
Profound depletion of hematopoietic cells in adult mice carrying an Evi1 exon3 deletion. (A) Kaplan-Meyer survival curves indicate significant lethality in Vav-iCre; Evi1^fl3/fl3^ mice, with a median survival of 7.7 weeks (Log rank test, Chi square p value <0.0001). (B) Hemograms for 6 to 9 week-old Vav-iCre; Evi1^fl3/fl3^ mice. These adult mice displayed leucopenia, severe anemia and thrombocytopenia. Mean ± SEM is indicated. *p<0.05, **p<0.01, ***p<0.001, unpaired t-test. (C) Flow cytometric profiles of bone marrow cells from Vav-iCre/+;Evi1^fl3/fl3^ and littermate control mice (Evi1^fl3/+^ or Evi1^fl3/fl3^). HSC and progenitor cell subpopulations were detected by a combination of markers (KSL: c-Kit^+^, S: Sca-1^+^, L: lineage^−^). We found a significant reduction of cells in Evi1-deleted samples, p = 0.00011 and p = 0.0024, for KSL and KL, respectively (unpaired t-test). (D) Colony forming counts for cells from bone marrow of Vav-iCre;Evi1^fl3/fl3^ and littermate control mice (Evi1^fl3/+^ or Evi1^fl3/fl3^). N = 3 for each group, p = 0.0019 (unpaired t-test). No BFU-E and CFU-Mix colonies were identified.

**Figure 4:**
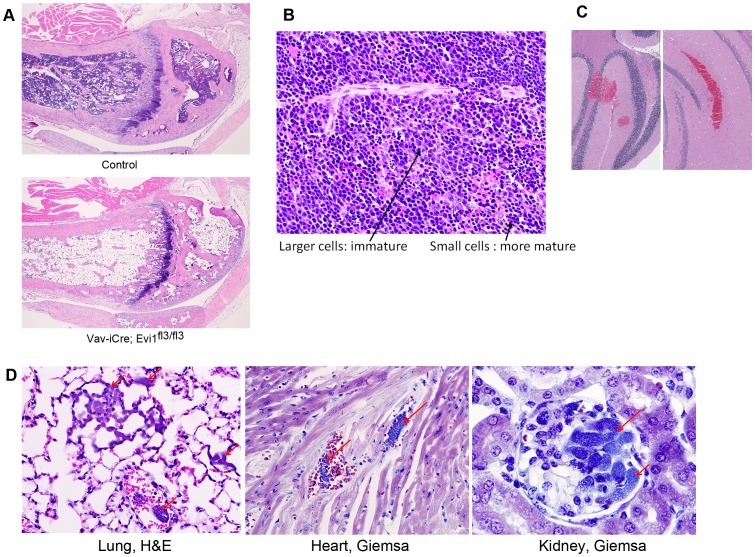
Spontaneous lethal bone marrow depletion in mice harboring an Evi1 exon3 deletion in the hematopoietic system. (A) Histology was performed on sick Vav-iCre; Evi1^fl3/fl3^ and littermate control mice. Bone marrow depletion was observed in the mutant mice. Adipose tissue replaced the hematopoietic cells in the bone marrow. (B) Increased erythropoiesis in the spleen of Vav-iCre; Evi1^fl3/fl3^ mice. No visible border was found between the red pulp and white pulp. Erythroid cells are shown by the arrows. Excess erythropoiesis in spleen likely happens to compensate for bone marrow loss. (C) H&E stained sections of the brain of a dying Vav-iCre; Evi1^fl3/fl3^ mouse. Hemorrhages (red areas) were visible at several locations (also see Fig. S3E in File S1). (D) Histological sections of tissues from dying Vav-iCre; Evi1^fl3/fl3^ animals showing bacteremia. Red arrows indicate the presence of bacteria in alveolar capillaries. Giemsa stains reveal the presence of cocci or small rods within glomerular capillaries. No sign of immune system defense (inflammatory cells) was observed despite the infection.

To further characterize the hematopoietic phenotype linked to the Evi1 exon3 deletion, we crossed Evi1^fl3/fl3^ animals with Vav-iCre transgenic mice [40]. Vav-iCre is expressed in all hematopoietic, but few other cell types, and as expected Vav-iCre/+, Evi1^fl3/fl3^ animals displayed a selective loss of Evi1 exon3 in the hematopoietic compartment (Fig. S3A in File S1). These mice did not die during prenatal development but instead died between 2.8 and 24.8 weeks of age (N = 37), with a median survival of 6.3 weeks (Fig. 3A). Heterozygous deletion of exon 3 did not affect the mortality rate compared to control mice (Fig. 3A). Most mice became weak and lost weight before dying (Fig. S3B in File S1). Hemograms were subsequently performed on Vav-iCre/+, Evi1^fl3/fl3^ weak animals and corresponding littermate controls +/+, Evi1^fl3/fl3^. The hematopoietic phenotype was dramatic, with severe thrombocytopenia, anemia and leucopenia in this conditional exon 3 deletion (Fig. 3B). Moreover, the number of KSL HSCs and KL progenitor cells in the bone marrow was close to zero (Fig. 3C). In addition, no colonies could be formed from Vav-iCre/+, Evi1^fl3/fl3^ bone marrow cells ex vivo (Fig. 3D). These results demonstrated a profound depletion of HSC and progenitor cells as well as downstream hematopoietic cells. Histological analysis of the bones of sick animals confirmed the spontaneous bone marrow hypoplasia (Fig. 4A), as hematopoietic cells were few or undetectable in the bone marrow cavity. This phenotype was accompanied by compensatory erythropoiesis in the spleen (Fig. 4B). Erythrophagocytosis with rosettes (Fig. S3C in File S1) was also identified in two animals, demonstrating immune perturbations. Bone marrow depletion can lead to hemorrhages due to lack of megakaryocytes and platelets. Indeed, bleeding in vital organs like the brain was observed in Vav-iCre/+, Evi1^fl3/fl3^ mice and was likely to be one major cause of lethality in these animals (Fig. 4C, S3D in File S1). Another major etiology was severe bacterial infections due to loss of immune defense. Gram-positive bacteria were found in the blood of the lungs, kidneys, and hearts of Vav-iCre/+, Evi1^fl3/fl3^ mice, indicating bacteremia (Fig. 4D). Collectively, these results describe a spontaneous lethal bone marrow failure upon deletion of Evi1 exon3 in the hematopoietic system. This hypomorphic phenotype is consistent with the profound HSC depletion seen in Evi1 exon 4 conditional knockout at E10.5–16.5 [12], but it occurs at a much later stage, in Evi1 exon 3 deleted adult mice.

### Congenital heart defects in Evi1^δex3/δex3^ newborn mice

**Figure 5:**
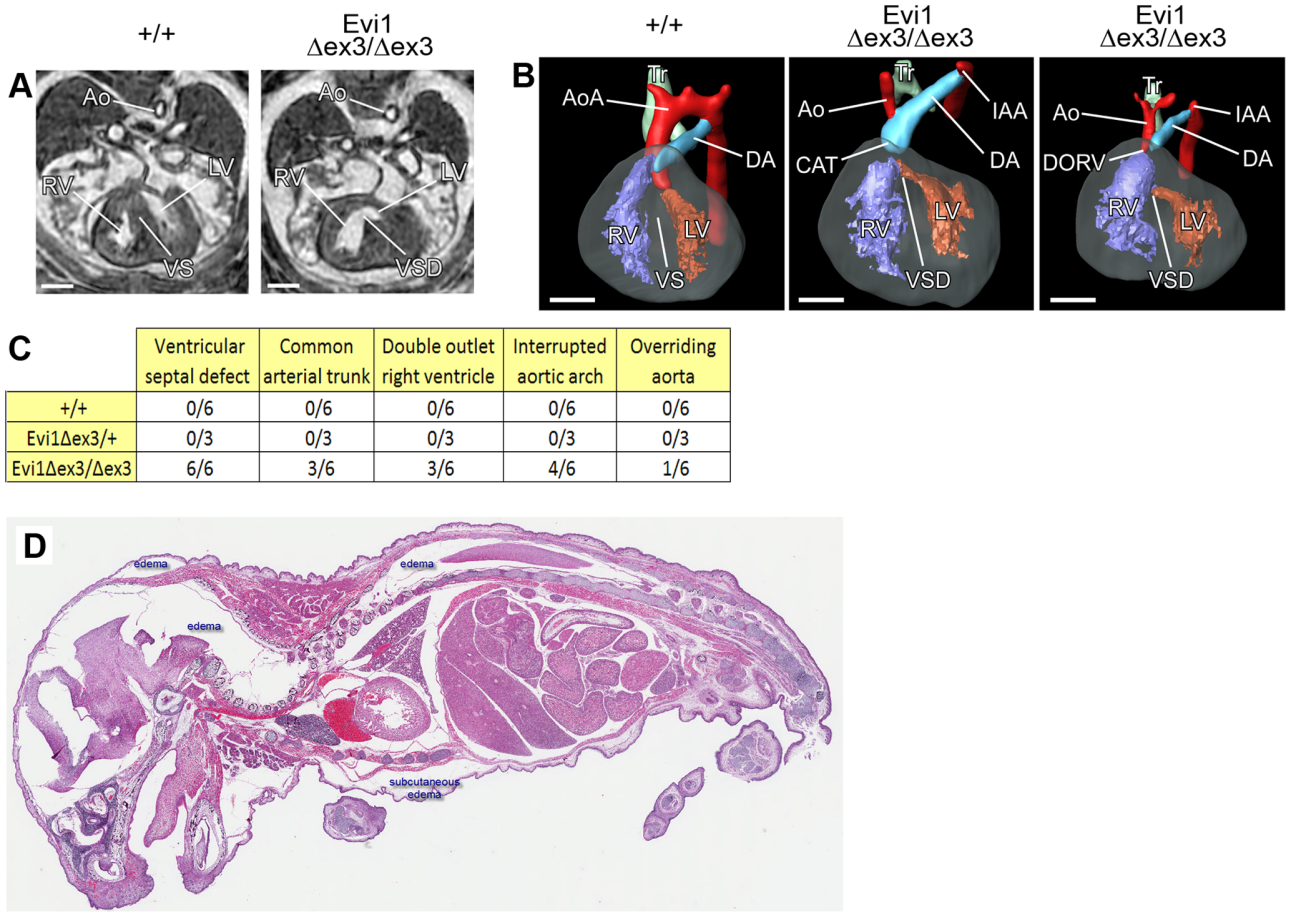
Cardiac malformations and failure in Evi1^δex3/δex3^ mice. (A) Transverse sections and (B) 3D reconstruction (left-ventral oblique view) of hearts from Evi1^δex3/δex3^ or wild type littermate (+/+) E15.5 embryos analyzed by magnetic resonance imaging (MRI). The aorta (Ao), right ventricle (RV), left ventricle (LV), ventricular septum (VS), trachea (Tr), aortic arch (AoA) and ductus arteriosus (DA) are indicated. Ventricular septal defect (VSD), interrupted aortic arch (IAA) and common arterial trunk (CAT) were observed in Evi1^δex3/δex3^ hearts. (C) List of the congenital heart defects identified in fifteen E15.5 embryos of various different genotypes by MRI and 3D reconstruction. (D) Hematoxylin and eosin staining of 5 µm sections of a sick Evi1^δex3/δex3^ pup. Subcutaneous and other tissue edema (white spaces) was present, consistent with heart failure.

Since it was unlikely that the perinatal lethality observed in Evi1^δex3/δex3^ mice was caused by the hematopoietic defects we looked for other possible causes. We used magnetic resonance imaging (MRI) to visualize organ formation in six Evi1^δex3/δex3^, three Evi1^δex3/+^ and six E15.5 control littermates, as previously described [41]. Structural abnormalities were observed in the hearts of all six Evi1^δex3/δex3^ embryos (Fig. 5A,B), while small benign bilateral cysts were observed in the jugular lymphatic sacks of two Evi1^δex3/δex3^ embryos (Fig. S4 in File S1). No defects were observed in wild type or heterozygous mutant animals. Evi1^δex3/δex3^ embryos displayed several congenital heart defects (Fig. 5C).

All six Evi1^δex3/δex3^ embryos had ventricular septal defects (VSD) - failure to form the septum between the ventricles of the heart (Fig. 5B,C).

Common arterial trunk (CAT), where two great arteries fail to separate and leave the heart as one common vessel, was also observed in 3 out of 6 Evi1^δex3/δex3^ embryos. Double outlet right ventricle (DORV), where both the aorta and pulmonary trunk leave one ventricle, was also observed in half of the Evi1^δex3/δex3^ embryos (Fig. 5B,C). In addition, overriding aorta (aorta originating just above the VSD) was seen in one Evi1^δex3/δex3^ embryo. Finally, aortic arch formation impairments were found in 4 out of 6 Evi1^δex3/δex3^ embryos (Fig. 5B,C). These impairments were manifested as an interrupted aortic arch (IAA), with a complete discontinuation between the ascending and descending parts of the aorta. These type of congenital heart defects are known to be viable *in utero* but lethal during the neonatal phase of life for other mouse knockouts [42], and thus likely represent the major cause of the perinatal lethality seen in Evi1^δex3/δex3^ pups. Consistent with this, heart failure was sometimes accompanied by oedema and congested lungs in Evi1^δex3/δex3^ pups (Fig. 5D).

### Mecom expression in the developing heart

**Figure 6:**
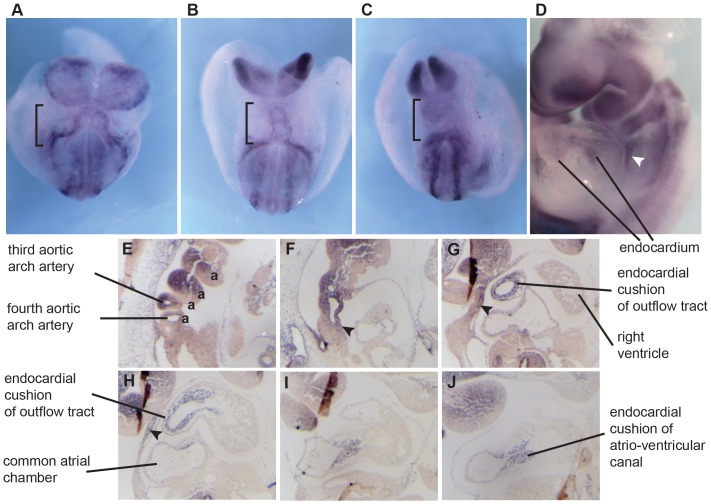
Expression of Mecom mRNA in cardiac structures of wild type embryos. (A–D) Whole mount mRNA *in situ* hybridization to show Mecom expression. A–C) Expression during subsequent stages of heart tube formation E8.5 (black brackets). D) At E9.5 Evi1 is expressed in the endothelial cells and in the endocardium of the heart and in the mesenchyme of the aortic arches. Expression also includes a population of migrating neural crest cells (white arrowhead). E–J) E10.5 Sagittal sections (from right to left) showing Evi1 in the aortic arches (a), mesenchyme of the secondary heart field (black arrowheads), outflow and atrio-ventricular canal endocardium including the cushions.

We next examined *Mecom* expression by mRNA *in situ* hybridization. At E8.5 *Mecom* was expressed in the forming heart tube (Fig 6A-C). By E9.5-E10.5 *Mecom* expression could clearly be localized to the endothelial cells and in the endocardium (Fig. 6D–J), and its expression was strong in the cushions of the atrio-ventricular canal (AVC). In the outflow tract, *Mecom* was not clearly expressed in the myocardium outer layer, but rather in the mesenchyme cells that are composed of cardiac neural crest. There was also clear expression in the neural crest cells which generates the majority of mesenchyme of aortic arches 1 and 2 (Fig 6E). We also saw *Mecom* in the stream of neural crest cells situated behind the heart (Fig 6D, arrowhead). Finally, there was additional *Mecom* signal in the mesenchyme cells of the secondary heart field (Fig 6E,F).Overall, we found that *Mecom* expression overlaps with the key cell populations in which defects could lead to the heart malformations we have described, especially the endocardium, the endocardial cushions, and the neural crest cells [42], [43]

### Evi1 controls the expression of genes that regulate heart development

**Figure 7:**
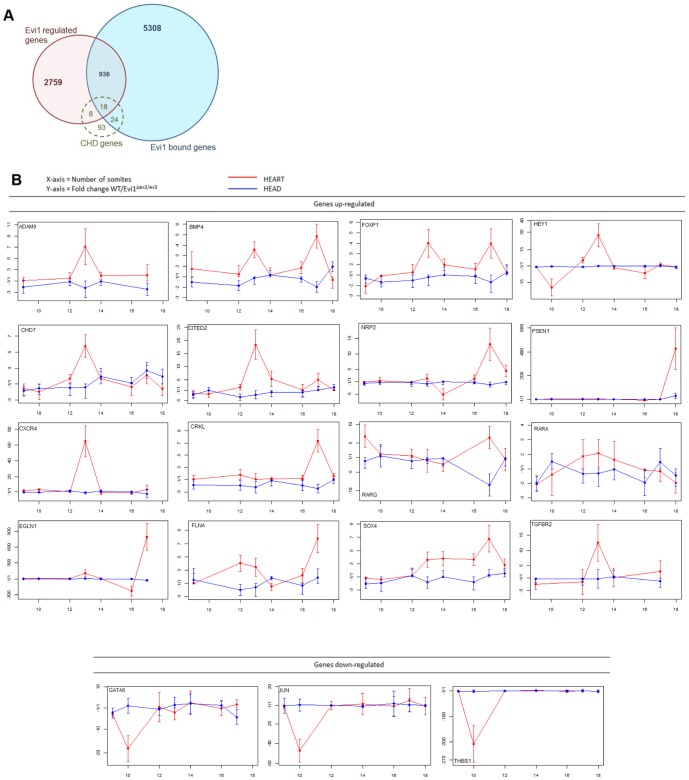
Evi1 regulates the expression of other CHD genes during embryonic heart development. (A) The number of CHD genes represented in Evi1 ChIP-Seq data (Evi1 bound genes) or in the list of genes regulated by Mecom. An enriched number of CHD genes were found bound or regulated by Mecom (50 out of 143 genes), p = 0.0453 and p = 0.0276, respectively. These genes represent potential Mecom target genes in heart development. (B) Mecom regulates the expression of 23 CHD genes, which contain Evi1-binding sites specifically in heart. Heart and head (neural crest) tissues were harvested from WT and Evi1^δex3/δex3^ embryos of somite number 9 to 18. RT-qPCR assays were performed. Genes considered to be mis-regulated in Evi1^δex3/δex3^ hearts were increased or decreased in expression by at least three fold in average for all samples of the same time-point. These graphs are representative of two to five independent experiments.

How might Evi1 act to control heart development? Because Evi1 is a transcription factor that can both activate or repress its target genes [16] we hypothesized that it might be part of the transcriptional program that controls heart development. To determine this, we searched the Mouse Genome Informatics (MGI) database [44] and found 143 Congenital Heart Defect (CHD) genes whose mutant heart phenotypes were similar to those observed in Evi1^δex3/δex3^ mice (Table S1). These genes were linked to the MGI Mammalian Phenotype identifications MP:0010402 (VSD), MP:0002633 (persistent truncus arteriosis, another name for CAT), MP:0000284 (DORV), MP:0004157 (IAA), and MP:0000273 (overriding aorta) [45]. We cross-compared these 143 genes with available EVI1 ChIP-Seq and differential microarray data [16]. Forty-two of these 143 genes contain known EVI1-binding sites, which constituted a significant enrichment (p = 0.0453, Chi-square with Yates correction), suggesting them as possible Evi1-target genes in heart (Fig. 7A). Similarly, the expression of 26 genes is known to be affected by Evi1 siRNA knock-down in SKOV3 cells (significant enrichment, p = 0.0276, Chi-square with Yates correction) [16], while 18 genes contain known Evi1-binding sites and are also effected by Evi1 siRNA knockdown (Fig. 7A, Table 1). This represents a very significant enrichment of CHD genes in Evi1 direct target genes (p<0.0001, Chi-square with Yates correction), strongly suggesting a functional involvement of these EVI1 target genes in heart development.

**Table 1:** List of 23 congenital heart defect (CHD) genes whose expression is disrupted in Evi1^δex3/δex3^ developing hearts.

mouse gene symbol	human gene symbol	common arterial trunk (MP:0002633)	Ventricular septal defect (MP:0010402)	double outlet right ventricle (MP:0000284)	overriding aorta (MP:0000273)	interrupted aortic arch (MP:0004157)	EVI1 target gene by ChIP-Seq	Regulated by EVI1 (microarray in SKOV3 cells)	Evi1^δex3/δex3^ affects gene expression in embryos hearts
Adam9	ADAM9		yes	yes			yes	up-regulated	up-regulated
Bmp4	BMP4	yes	yes	yes		yes	yes		up-regulated
Bmpr2	BMPR2					yes	yes		up-regulated
Cav1	CAV1						yes	down-regulated	up-regulated
Chd7	CHD7		yes			yes	yes		up-regulated
Cited2	CITED2	yes	yes	yes	yes	yes	yes		up-regulated
Crkl	CRKL		yes	yes	yes	yes	yes		up-regulated
Cxcr4	CXCR4		yes				yes	down-regulated	up-regulated
Egln1	EGLN1	yes	yes			yes	yes	down-regulated	up-regulated
Flna	FLNA	yes	yes	yes		yes	yes		up-regulated
Foxp1	FOXP1	yes	yes	yes			yes		up-regulated
Gata6	GATA6	yes	yes	yes		yes	yes		down-regulated
Hey1	HEY1	yes	yes				yes	up-regulated	up-regulated
Jag1	JAG1				yes		yes	up-regulated	up-regulated
Jun	JUN	yes					yes	down-regulated	down-regulated
Nf1	NF1	yes		yes			yes		up-regulated
Nrp2	NRP2	yes					yes	up-regulated	up-regulated
Psen1	PSEN1			yes			yes	down-regulated	up-regulated
Rarg	RARG	yes		yes			yes	up-regulated	up-regulated
Rxra	RXRA	yes		yes			yes		up-regulated
Sox4	SOX4	yes				yes	yes	up-regulated	up-regulated
Tgfbr2	TGFBR2					yes	yes	up-regulated	up-regulated
Thbs1	THBS1	yes	yes				yes	down-regulated	down-regulated

These genes were previously found targeted by Evi1 in ChIP-Seq and microarray experiments [16], indicating they may be directly regulated by Evi1.

These computational comparative analyses have provided a list of 50 genes that are likely to be enriched for genes that are regulated by Evi1 during heart development (Table S1, Figure 7A). To provide additional evidence for this, we dissected hearts, and heads as a control, from a range of Evi1^δex3/δex3^ embryos between E8 and E10, in order to determine if these candidates are deregulated due to the disruption of *Mecom* activity.

We extracted mRNA from mutant and wild-type embryonic hearts and heads, and performed reverse transcription (RT) and qPCR to quantitate the level of expression of 31 of the Evi1 candidate target genes (Fig. 7B, S5). Due to limited amount of RNA from embryonic heart, we chose to assess the 18 CHD genes previously found occupied and regulated by Evi1, plus 14 CHD genes bound by Evi1. We then used the 2^−ÄÄCt^ method [30] to calculate the fold change in expression between wild type and mutant embryos. We found that the Evi1 exon3 deletion had no effect on the expression of eight genes (Fig. S5 in File S1), while three were downregulated and 20 were upregulated in expression in *Mecom* mutant hearts (Table1, Fig. 7B). This was consistent with MECOM being a known dynamic modulator of transcription that can either activate or repress genes, depending on the recruitment of coactivators or corepressors [46].Of the 13 genes regulated by Evi1 both in cardiac development and in SKOV3 ovarian carcinoma cells, 9 genes showed Evi1-mediated changes in expression level in a similar manner (Jun, Thbs1, Adam9, Hey1, Jag1, Nrp2, Rarg, Sox4, and Tgfbr2). Some of these regulatory relationships were also consistent with previous reports. For instance, in cell line models, Jun expression was found up-regulated by Evi1 through its direct binding to Jun promoter [16], [47]–[50]. The Sox4 transcription factor and Evi1 cooperate to induce myeloid leukemia [51]; and Evi1 was shown to bind to Sox4 promoter and regulate its gene expression [16], providing evidence of transactivation of Sox4 by Evi1. Collectively, these results demonstrate that Evi1 modulates, in embryonic heart, the expression of genes that are important for controlling heart development.

We also performed a literature search to compare the gene expression patterns of these Mecom-deregulated factors to the *Mecom* embryonic heart expression pattern we describe (Fig.6). This analysis (Table 2) confirmed common expression in the endocardium and endocardial cushions, as well as in the aortic arches and outflow tract - especially in the neural crest cells.

**Table 2:** Overview of Major Reported Expression Domains.

Gene	Reported Expression Domains	References
**Mecom**	**AA, CC/HT, End+Csn, NC, SHF**	
Adam9	End+Csn, Myo	[56], [57]
Bmp4	AA, Myo, NC, OFT, SHF	[58], [59], [60]
Bmpr2	AA, End, Myo, NC	[58], [59], [60]
Cav1	End	[61]
Chd7	AA	[62]
Cited2	AA, CC/HT, End+Csn, Myo, OFT	[63], [64]
Crkl	AA, NC	[65]
Cxcr4	AA, Myo	[66]
Flna	AA, End+Csn, NC, OFT	[67]
Foxp1	End+Csn, Myo, OFT	[68]
Gata6	End+Csn, Myo, OFT, NC	[69], [70]
Hey1	AA, End, OFT	[71], [72], [73]
Jag1	AA, End, OFT	[71], [74]
Jun	AA, End+Csn, OFT, SHF	[75]
Nf1	AA, End+Csn, Myo, NC	[76]
Nrp2	NC	[77], [78]
Psen1	AA, End+Csn, Myo, NC, OFT	[79], [80]
Rarg	AA	[81]
Rxra	AA, End+Csn, Myo, NC, OFT	[82]
Sox4	End+Csn, Myo	[68], [83]
Tgfbr2	AA, CC/HT, End+Csn, Myo, NC	[84], [85], [86]
Thbs1	End, Myo	[87]

**Key**

AA – Aortic Arch and Aortic Arch Arteries.

CC/HT - Cardiac Crescent/Heart Tube.

End – Endocardium (+Csn – including Cushions).

Myo – Myocardium.

NC –Neural Crest (Cardiac).

OFT – Outflow Tract.

SHF – Secondary Heart Field.

## Discussion

Our results demonstrate that deletion of Evi1 exon 3 produces a hypomorphic allele compared to previous studies involving Evi1 exons 4 and 7, where their removal produced complete null alleles [12], [29]. Deletion of exon 3 indeed does not affect Evi1, Evi1δ105 [37] and Evi1δ324 protein production but does block the generation of Mds1-Evi1 protein production. All Evi1 isoform proteins expressed in these mice are expected to carry a 42 amino acid truncation at the N-terminus that constitutes nearly 4% of the protein. Such truncated proteins would be predicted to lack one zinc finger motif out of the seven present in the proximal DNA-binding site. It is not completely clear if and how this truncation affects Evi1 transcriptional activity or function. Several findings suggest that translation from Evi1 exon4 ATG start site produces a functional protein. First, the exon4 contains the best Kozak sequence with highest cross-species conservation. Thus, it is possible that the exon4 translation start site may be naturally produced in vivo. Secondly, a previous study has suggested that Evi1 protein initiated from exon 4 is oncogenic and able to give rise to leukemic clones in mice [52]. Retroviral insertional mutagenesis screens in mice have identified Evi1 isoform as a targeted mutant gene in myeloid leukemia [53], [54]. Sequencing of the retroviral insertion sites from these tumors has shown that the majority of insertions are located upstream of Evi1 coding sequence, where they serve to upregulate the expression of oncogenic Evi1 but block the expression of Mds1-Evi1. The genomic region located between exons 3 and 4 is only 4 kb compared to the rest of the Evi1 upstream region which is 90 kb in size, thus providing 23 times less chance to contain a retrovirus insertion by random chance. However, retroviral insertions located between exon 3 and 4 have been described in tumors, which would serve to activate Evi1 translation from the alternative translation start site located in exon 4 [52].

The profound embryonic lethal disruption of HSC renewal seen in other studies [12], [13] was not present in our Evi1^δex3/δex3^ mutant embryos and newborn pups. However, we did identify a dramatic perturbation of hematopoietic repopulation activity in Vav-iCre/+, Evi1^fl3/fl3^ young adult mice. To our knowledge, there is no current genetically-modified mouse model that mimics spontaneous bone marrow failure as seen in the Vav-iCre/+, Evi1^fl3/fl3^ mice. They therefore constitute the first model of spontaneous lethal bone marrow failure in the adult. Surprisingly, the hypomorphic deletion of Evi1 could delay the phenotype of hematopoietic failure and the appearance of bone marrow depletion. This in is line with a previous study [21] that specifically implicated Mds1-Evi1 in the regulation of long term HSC repopulating activity [55] and Evi1 in short term HSC renewal activity [12], [29].

The delay in acquisition of the hematological phenotype in Evi1^δex3/δex3^ knockout mice allowed the embryos to survive to the perinatal period and the congenital heart defects found in these mice to be observed. Our results are also consistent with those reported for Evi1 exon 7 knockout mice published in 1997, which reported that E10.5 Evi1^-/-^ mutant embryos displayed heart failure. Although their data based on only one histology section are not clear, Evi1^δex7/δex7^ knockout embryos were reported to display arrested heart development with a looping defect of the posterior part of the heart and a poorly developed constriction between atria and ventricle [29], which is different from our findings. At the time of this previous study, the technologies to study embryonic cardiac development were based only on histological methods, which could not allow precise interpretations of the pathology. In our studies we used MRI and 3D modeling to clearly define the pathology and heart developmental defects in Evi1 exon 3 knockout embryos.

We provide evidence that Mecom belongs to a transcriptional regulatory network that controls heart development. Mecom expression overlaps with the expression of multiple other factors required to form the heart (Table 2). These factors can be Mecom targets, and their expression is deregulated expression in the Evi1^δex3/δex3^ mutant heart. Of particular interest may be factors in the Notch and TGFβ pathways as that Mecom or its homologues interact with these pathways [22]. In the endocardium for example, there is clear overlap of Mecom with the Notch ligand Jag1 and the TGFβ receptor Tgfbr2.

The endocardium is major site of Mecom expression in the heart, and it is possible that Mecom regulates gene expression directly in this tissue. The cushions cells of the AVC originate from endocardium via an epithelial–mesenchymal transition, and they form the partition between the ventricles and the atria (atrio-ventricular canal and later valves). This partition provides the matrix for the growing ventricular and atrial septa [42], [43]. Another possible site of Mecom action is in the neural crest cells. The spectrum of phenotypes seen in the Evi1^δex3/δex3^ knockout heart could also be attributed to defects in these cells causing disrupted remodelling of the aortic arches, and to a failure to septate the outflow tract [43]. Further studies (perhaps using a floxed-*Evi1* null allele [12] and specific Cre lines) can be used address if Mecom is required in a particular heart cell population, or in multiple populations to drive heart development.

## Supporting Information

File S1 **Figure S1, Targeting and knockout of Evi1 exon3. Figure S2, An alternative protein translation site located in Evi1 exon 4 and structure of the translated protein. Figure S3, Deletion of Evi1 exon 3 in the hematopoietic compartment. Figure S4, Small bilateral cysts in jugular lymphatic sacks of Evi1^δex3/δex3^ embryos. Figure S5, CHD gene expression in Evi1^δex3/δex3^ embryos.**
(DOCX)

Table S1 **List of 143 congenital heart defect genes with similar heart phenotypes as thosed observed in Evi1^δex3/δex3^ mice.** All 143 genes linked to the Mammalian Phenotype identifications MP:0010402 (VSD), MP:0002633 (persistent truncus arteriosis, other name for CAT), MP:0000284 (DORV), MP:0004157 (IAA), MP:0000273 (overriding aorta) in the MGI database [88]. The genes found in previous Evi1 ChIP-Seq and microarray experiments [89] provide potential Mecom target genes in heart development.
(XLSX)
