# Supplementary material for: Mice Carrying a Hypomorphic Evi1 Allele Are Embryonic Viable but Exhibit Severe Congenital Heart Defects
Source: PLoS One. 2014 Feb 27;9(2):e89397. doi: 10.1371/journal.pone.0089397 (PMC3937339; doi:10.1371/journal.pone.0089397)
Supplement: File S1 — Figure S1, Targeting and knockout of Evi1 exon3. Figure S2, An alternative protein translation site located in Evi1 exon 4 and structure of the translated protein. Figure S3, Deletion of Evi1 exon 3 in the hematopoietic compartment. Figure S4, Small bilateral cysts in jugular lymphatic sacks of Evi1δex3/δex3 embryos. Figure S5, CHD gene expression in Evi1δex3/δex3 embryos. (DOCX) [file pone.0089397.s001.docx]

Supplemental Information

**File S1**

### ****Supplementary experimental procedures****

*Primers for Quantitative Real-Time RT-PCR (qRT-PCR).*

| Gene symbol | exons | Forward primer | Reverse primer |
| --- | --- | --- | --- |
| Evi1 | 2, 3 | GCTATGATCAGCACAACCTTGTTG | TGTCTGCGACTACTCGGTAGAATATC |
| Evi1 | 3, 4 | ATGGCGCCTGACATCCA | GGATTCAAAGAGCTGGTCACAGT |
| Evi1 | 13, 14 | CCCGGTGAGGTATAAAGAGGAA | ATCTGTGAAGTGCCTTATGTGATCTAG |
| Tubg1 | 6, 7 | ACAACTCCCTCCTCACACTAAAGAG | TGTCCAGCACCACCACACA |
| Adam19 | 6, 7 | CATCGAGCCCGTCCCTAAC | GCGTGAGATGTTCGGATCTGT |
| Adam9 | 6, 7 | CTCTGCACAACAGCTCACACTTT | TCTCAGAGGCTCCTGGTGGAT |
| Ate1 | 5, 6 | AGGATGCTGTTGACGGTGACT | GGTCACTGAGTGTTTTGAGATCACA |
| Bmp1 | 14, 15 | CTCCCAACAAAAACTGCATCTG | AATTGCAGGGAGATACGGTACTG |
| Bmp4 | 3, 4 | CGAGCCATGCTAGTTTGATACCT | CCCTGAATCTCGGCGACTT |
| Bmpr2 | 2, 3 | TCTTGGGATAGGTGAGAGTCGAA | CGTGCTCCCTTTGGAACATAA |
| Cav1 | 2, 3 | CAACATCTACAAGCCCAACAACA | GCGCGTCATACACTTGCTTCT |
| Chd7 | 3, 4 | CCAGGTCAGCGCACAGAAC | TCTATCGACGGCAGGTAATCG |
| Cited2 | 2, 3 | GTCCCCGCGGCAATG | TCCTCGTCGATGAAATCAGTGT |
| Crkl | 1, 2 | TGTCTCGCACTACATCATCAACTC | TCCTGGTCCCCGATCTTAAA |
| Cxcr4 | 2, 3 | TTACCCCGATAGCCTGTGGAT | GCAGGACGAGACCCACCAT |
| Ece1 | 5, 6 | GTGTCTCAGTGACCAGCTCTATCC | GAAGTCCTGGCACGGGTCTA |
| Egln1 | 1, 2 | CGTTGCCCGCGTTGA | GCCGTGCTTGTTCATGCA |
| Flna | 3, 4 | GCAAGGCTATTGTGGATGGAA | CAGGATCAGGGTCCAGATGAG |
| Foxp1 | 5, 6 | TTCCCGTGTCAGTGGCTATG | GAGGATCTGCTGCATTTGTTGA |
| Gata6 | 4, 5 | ACGGCGGCTTGGACTGT | TTCTACGCCATAAGGTAGTGGTTGT |
| Hey1 | 5, 6 | GGAATGCCTGGCCGAAGT | TCGGAGGCATCGAGTCCTT |
| Jag1 | 2, 3 | CCGCGACGAGTGTGATACG | CAGTGACGCGGGACTGATACT |
| Jun | 1, 1 | CCGCCCCTGTCCCCTAT | TCCTCATGCGCTTCCTCTCT |
| Nf1 | 2, 3 | GCACCGAGCACAACAAGGA | CCCACTGATGACCAGAGAAAACT |
| Nrp1 | 2, 3 | TGTGGCGGGACCATAAAAA | GAGGGTAACCGGGAGATGTGA |
| Nrp2 | 5, 6 | GGGATCCTCTCCTTGACCTTTC | GTGCGGAGAAGCCATCCTT |
| Psen1 | 5, 6 | CCCTGCACTCGATCCTGAAT | GGACCACCAGGAGGATGGT |
| Ptpn11 | 2, 3 | CTGGTGTGGAGGCAGAGAATC | CTTGCTAAAAAACTGCCATCGA |
| Rara | 3, 4 | GCGGGCACCTCAATGG | GGAGAGTCCACCCAGCATAGG |
| Rarg | 4, 5 | CTCGCCCGACAGCTATGAAC | GCTTTGCTGACCTTGGTGATG |
| Rxra | 3, 4 | GAGGATATCAAGCCGCCACTAG | CTGAGGGATGGGCAGGAA |
| Sox4 | 1, 1 | CAAGCGGCTAGGCAAACG | CCTGGATGAACGGAATCTTGTC |
| Ssr1 | 4, 5 | GCTAGATGCCTCGTTCCGTTA | AGGAAGAGCTGTGAAATTCTGGAT |
| Tgfbr2 | 2, 3 | TGGCGGTGCGGTCAAG | CGCAAGTGGACAGTCTCACATC |
| Thbs1 | 3, 4 | GGCCGACAAAGGCTTCATC | GCCCCGGGTCTTCTTCAT |

### ****Supplementary Figures****

**
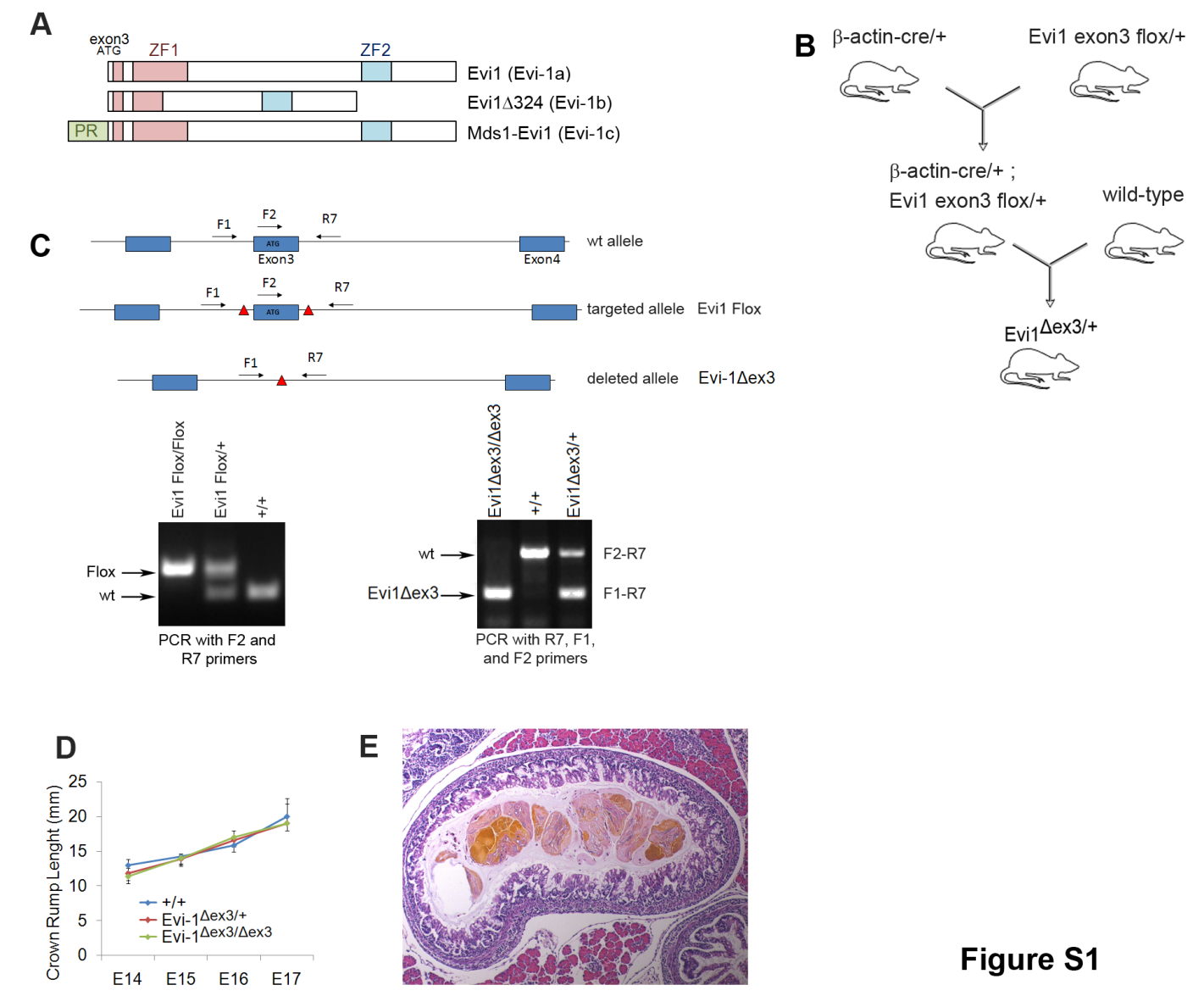
**

**Figure S1 Targeting and knockout of Evi1 exon3**

(A) Representation of the major Mecom isoforms. ZF1: zinc finger domain 1, ZF2: zinc finger domain 2, PR: PR domain. The Evi-1a protein product is also referred to as Evi1, Evi-1b protein as Evi1Δ324 and Evi-1c protein as Mds1-Evi1. (B) Mating scheme used to produce the germ line deletion of Evi1 exon3. Evi1 ^Δex3/+^ animals were generated by mating Evi1^fl3/+^ mice with β-actin-Cre transgenic mice. (C) Targeted deletion of Evi1 exon3 and PCR genotyping results to assess the presence of floxed exon 3 or the exon 3 deletion following genetic crosses. (D) Sizes of the embryos of various genotypes. Embryonic growth was not affected by the exon 3 deletion. (E) Haematoxylin and eosin staining of 5µm sections of an Evi1^Δex3/Δex3^ 28hr-old pup. The stomach contained meconium with digested milk.


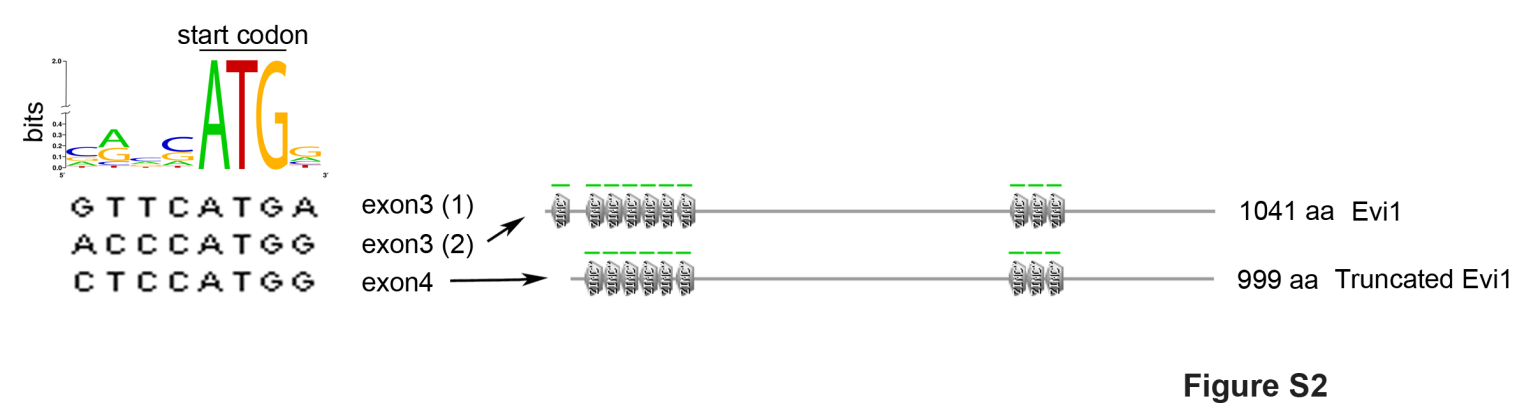


**Figure S2 An alternative protein translation site located in Evi1 exon 4 and structure of the translated protein**

The three ATG start sites located in Evi1 exons 3 and 4 are conserved Kozak consensus sequences [[1](#_ENREF_1)] and may therefore constitute authentic protein translational start sites. Translational initiation from the ATG start site located in exon 4 is predicted to produce a 999 amino acid protein with one zinc finger motif truncated in the N-terminal zinc finger domain.


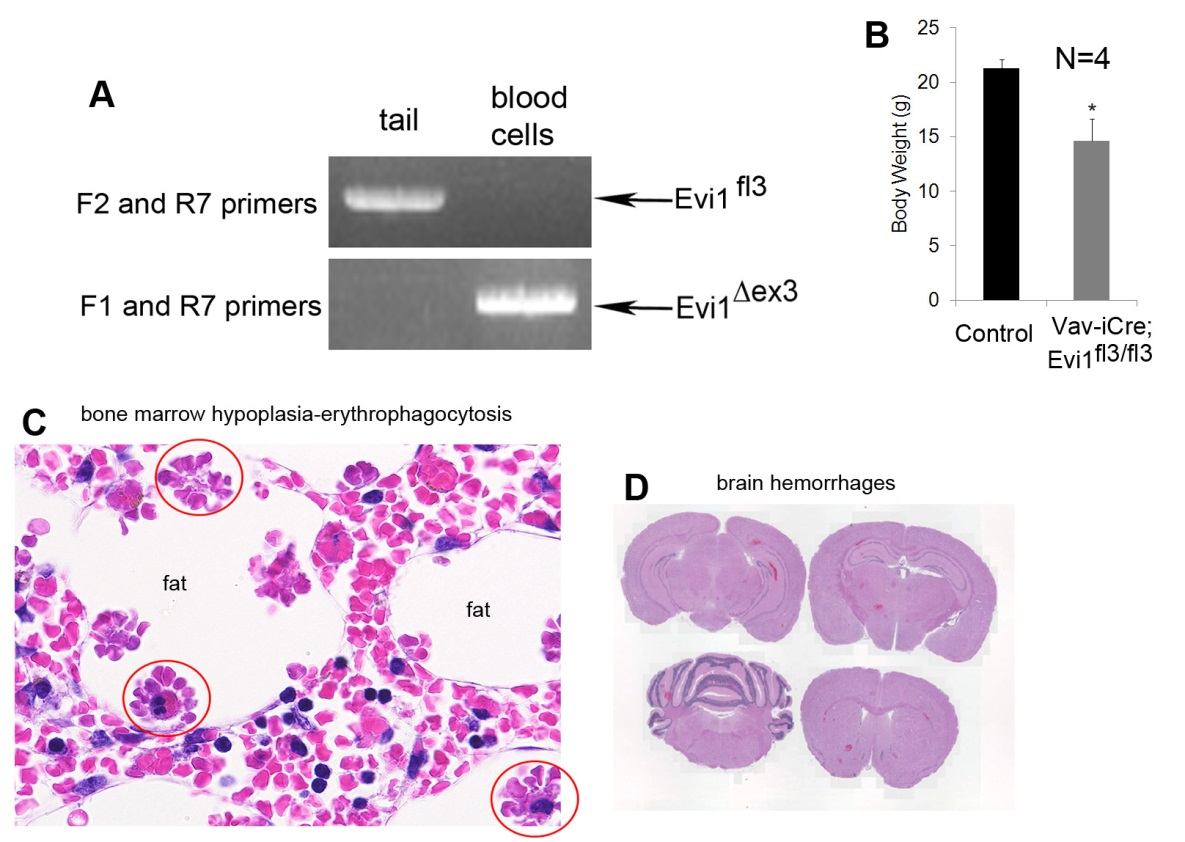


**Figure S3 Deletion of Evi1 exon 3 in the hematopoietic compartment**

(A) PCR of tail and blood cells from a Vav-iCre; Evi1^fl3/fl3^ healthy 3 week-old mouse to detect Evi1^fl3^ and Evi1^Δex3^ alleles (see Fig S1B for primer location at the Evi1 loci). Evi1 exon3 was deleted in blood cells but not in tail tissue. (B) Body weight loss in 6 to 9 week-old sick Vav-iCre; Evi1^fl3/fl3^ mice compared to littermate controls. N=4 for each group, *p=0.022, mean ± SEM. (C) Hypocellular bone marrow in a Vav-iCre; Evi1^fl3/fl3^ mouse. In vivo formation of macrophage-erythrocyte-rosettes (red circles) was observed when the erythrocytes adhered to the cell surface of macrophages to be phagocytosed. This lesion is commonly due to infection or autoimmune disease. (D) H&E staining of the brain of a comatose Vav-iCre; Evi1^fl3/fl3^ mouse. Hemorrhages (red foci) were visible at several locations (also see Fig. 4B)


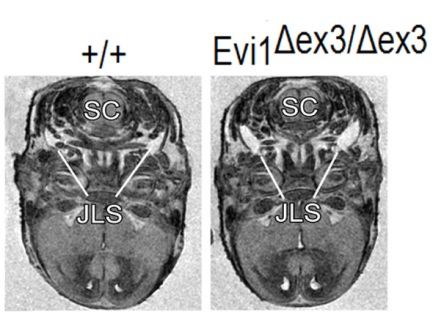


**Figure S4 Small bilateral cysts in jugular lymphatic sacks of Evi1^Δex3/Δex3^ embryos**

The only abnormality other than heart-related abnormalities identified by magnetic resonance imaging in Evi1^Δex3/Δex3^  mice were small bilateral cysts in the jugular lymphatic sacks (JLS). Spinal cord (SC).


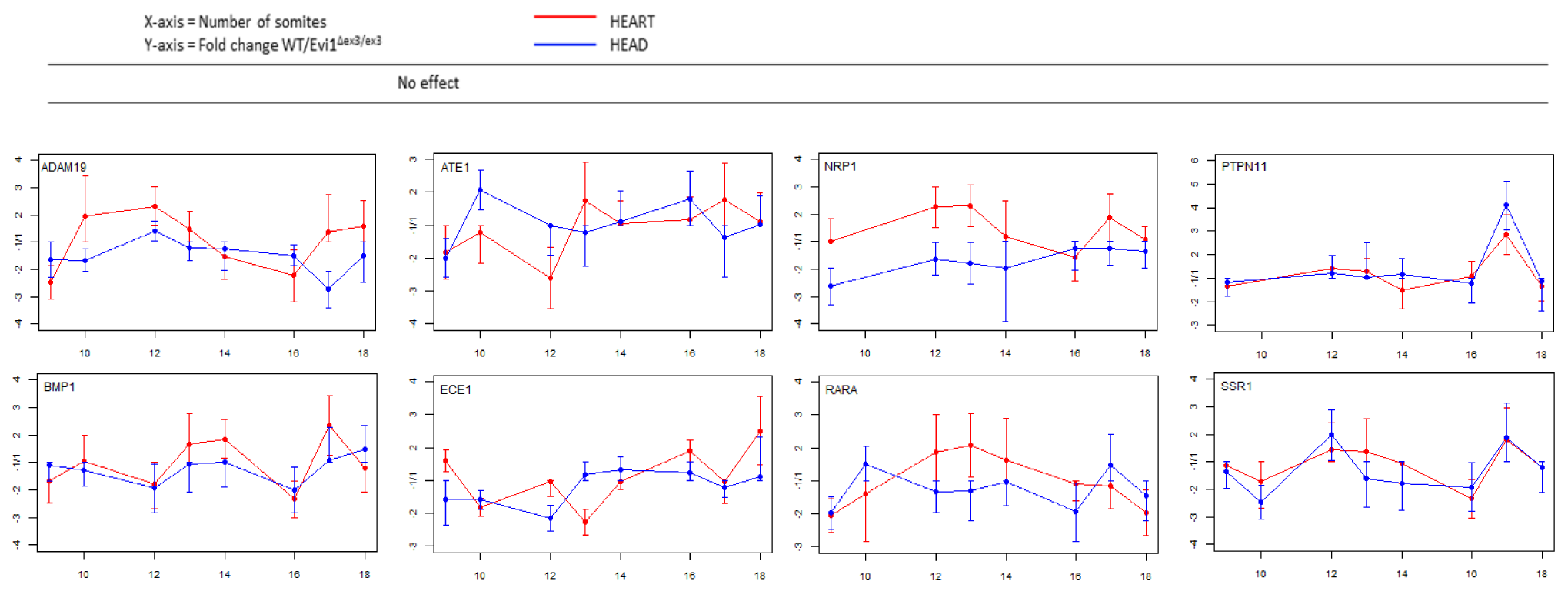


**Figure S5 CHD gene expression in Evi1^Δex3/Δex3^ embryos**

The expression of 32 CHD genes was assessed by RT-qPCR in heart and neural crest (head) tissues of wild type and Evi1 mutant embryos. The somites were precisely counted for each embryo before harvesting the heart and neural crest. The graphs are representative of at least two independent experiments. Here are shown the data from the genes for which Evi1^Δex3/Δex3^ had no significant effect on CHD gene expression.

**SUPPLEMENTAL REFERENCES**

1. Harhay GP, Sonstegard TS, Keele JW, Heaton MP, Clawson ML, et al. (2005) Characterization of 954 bovine full-CDS cDNA sequences. BMC Genomics 6: 166.
